# De novo glioblastoma in the territory of a recent middle cerebral artery infarction and a residual meningioma: pathogenesis revisited

**DOI:** 10.1186/s12957-016-0876-7

**Published:** 2016-04-18

**Authors:** Waseem Yaghmour, Maher E. Kurdi, Saleh S. Baeesa

**Affiliations:** Division of Neurosurgery, Faculty of Medicine, King Abdulaziz University, P.O. Box 80215, Jeddah, 21589 Kingdom of Saudi Arabia; Department of Pathology, King Abdulaziz University, Jeddah, 21589 Kingdom of Saudi Arabia

**Keywords:** Glioblastoma, Meningioma, Infarction, Gliosis, Pathogenesis

## Abstract

**Background:**

The pathogenesis of glioblastoma is complex, and the implicated molecular mechanisms are yet to be understood. There are scattered reports describing a possible relationship between meningioma and glioblastoma and more rarely a relationship between infarction and glioblastoma.

**Case presentation:**

We are reporting a 32-year-old male who developed left middle cerebral artery (MCA) infarction as a surgical complication for sphenoid meningioma. He developed recurrent symptoms 4 months later due to development of a glioblastoma adjacent to both the territory of the prior MCA infarct and the residual meningioma.

**Conclusions:**

This case adds further contribution to the literature of the possible pathological association between glioblastoma and brain infarction on a background of meningioma.

## Background

Several authors have reported the development of glioblastoma in areas of reactive gliosis [1]. Others have described both synchronously and, less commonly, asynchronously presenting meningioma and glioblastoma [2–4]. Studies on the relationship between glioma and traumatic brain injury have been conducted [5, 6].

Furthermore, there are very few reports relating strokes to glioblastoma, and most of them describe infarctions secondary to glioblastoma [7, 8], while the development of glioblastoma in the territories of previous cerebral infarctions has been rarely reported [1, 9]. Many hypotheses have been suggested to explain these observations, yet they remain speculative in nature and the glioblastoma developmental process is still obscure and not clearly understood [1–3]. We discuss two possible theories of glioblastoma development in such cases: first is the pathogenic mechanism of coexisted glioblastoma and meningioma and second is the development of glioblastoma secondary to the middle cerebral artery (MCA) infarct. The uncovering of the mechanisms that led to the phenomena mentioned above could further our knowledge regarding the pathogenesis of glioblastoma and subsequently their management.

We are reporting a 32-year-old male who underwent surgical resection of a left sphenoid meningioma that was complicated by an iatrogenic injury of the MCA with subsequent infarction. The patient had a remarkable recovery from the stroke but deteriorated 6 months later; radiological and histopathological examination revealed that he developed a glioblastoma in the territory of the previous infarction. The literature concerning glioblastoma developmental process is reviewed.

## Case presentation

**Fig. 1:**
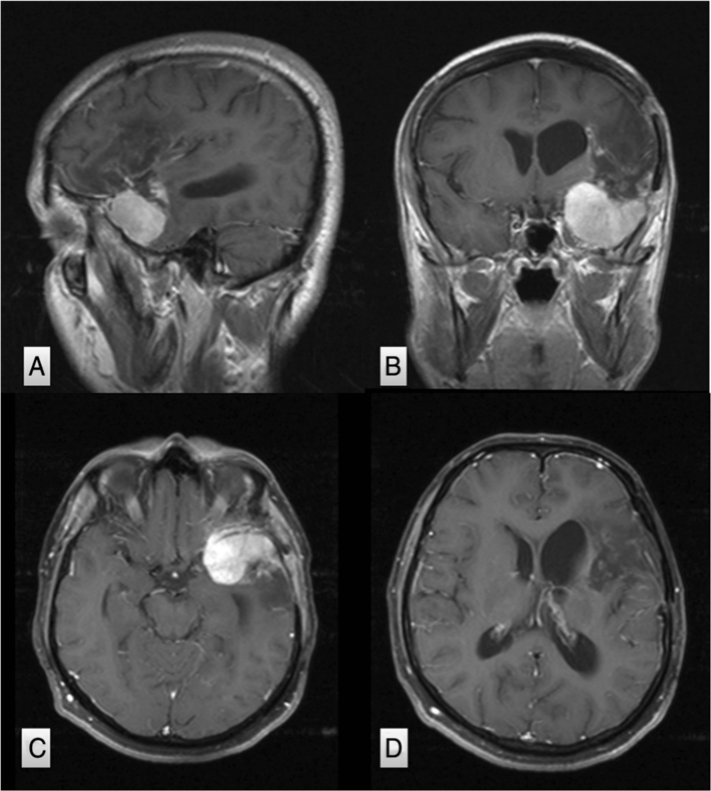
Post-contrast parasagittal (**a**), coronal (**b**), and axial (**c**, **d**) MRI scans performed 3 weeks after left frontal-temporal craniotomy demonstrating significant residual enhancing left sphenoid wing meningioma and ischemic changes in the left MCA territory

**Fig. 2:**
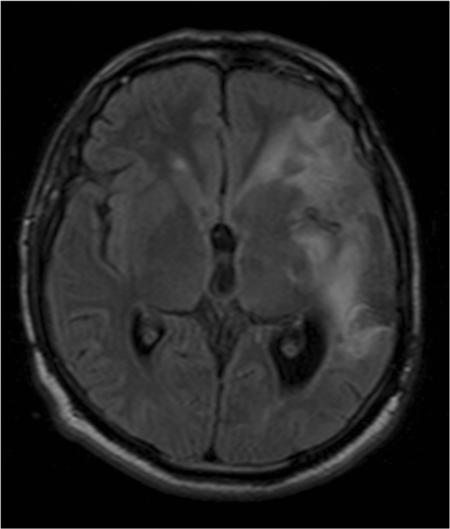
The extent of the infarction was demonstrated by FLAIR MRI sequence

**Fig. 3:**
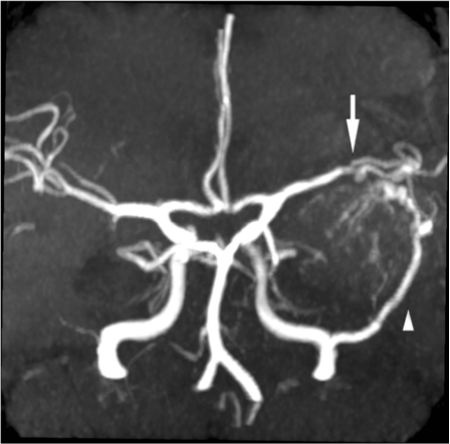
MR angiographic scan reveled marked decreased flow in the left MCA (*arrow*) and prominent tumor supply from the middle meningeal artery (*arrowhead*)

A 32-year-old male first presented in January 2010 with a progressive headache for a 4-month duration. He was investigated with computed tomography (CT) and magnetic resonance imaging (MRI) scans that revealed large left sphenoid wing meningioma. He underwent left frontal craniotomy in a hospital at a neighboring country, for resection of the sphenoid wing meningioma. Brain swelling complicated the attempt of tumor resection; as a result, surgery was terminated after partial resection of the tumor. The patient had a complicated postoperative course with development of left middle cerebral artery ischemia causing aphasia and right dense hemiplegia. He was transferred to King Abdulaziz University hospital 3 weeks after surgery for further management. The pathology report from his referring hospital revealed that the tumor specimen of what has been resected was consistent with WHO grade I meningioma. On admission, he was conscious and alert but with marked expressive aphasia, upper motor right facial weakness, and power of grade 2 right-side hemiparesis. Routine laboratory investigation, including hematology, electrolytes, and renal and coagulation profiles, were within normal limits. MRI scans of the brain revealed significant residual meningioma of the left sphenoid wing meningioma (Fig. 1). There was a left cerebral infarction demonstrated on the fluid-attenuated inversion recovery (FLAIR) MRI scans (Fig. 2). The MCA was partially narrowed at the bifurcation; there was still significant tumor blood supply from the middle meningeal artery (Fig. 3). The patient was evaluated by the neurology team who started him on antiplatelet medication (aspirin, 80 mg daily) and advised delaying surgery 8–12 weeks to allow further recovery from stroke. He was transferred to the rehabilitation center where he received an extensive speech and physical therapy for 3 months with subsequent significant neurological improvement. He remained with only mild right-hand weakness of grade 3, and subtle word-finding difficulty, and an elective admission was planned for resection of the residual meningioma.

**Fig. 4:**
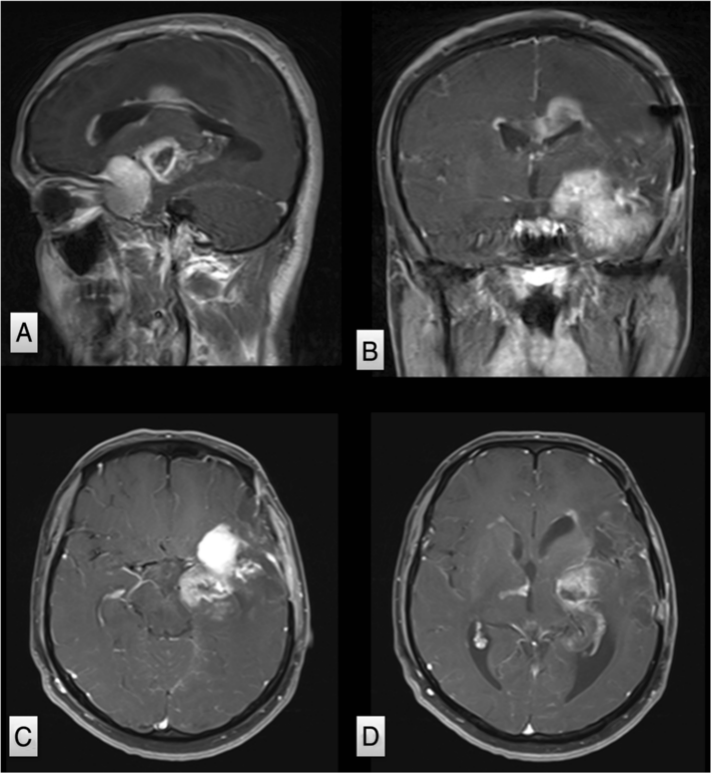
MRI study performed 5 months from the first surgery. Post-contrast parasagittal (**a**), coronal (**b**), and axial (**c**, **d**) MRI scans were performed demonstrating no change in the size of the meningioma, but there are multiple ring enhancing lesions in the medial temporal lobe adjacent to the meningioma, and in the infarcted tissue involving the corpus callosum

**Fig. 5:**
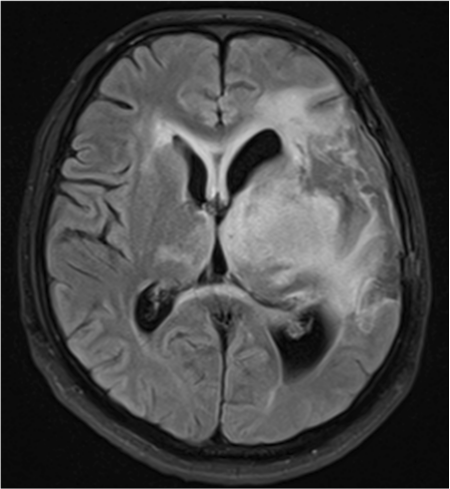
The extent of the new lesions in the left hemisphere (frontal, temporal, parietal region) was demonstrated by FLAIR MRI sequence with subependymal infiltration crossing to the right hemisphere

His second presentation to the emergency department, 3 weeks prior his scheduled admission, was with progressive headache over 2 weeks. He was confused with worsened speech and right hemiparesis. Brain MRI scan revealed unchanged size of the residual meningioma and the previous infarction, but there were new enhancing multi-focal and multi-centric deep frontotemporal lesions within and adjacent to the infarcted region and adjacent to the residual meningioma (Fig. 4). The extent of the edema and the new tumor infiltration was demonstrated by FLAIR MRI scan which involved the left hemisphere and extended to the right side as well (Fig. 5). The patient was admitted and started on steroids and had a stereotactic biopsy of the enhancing part of the new frontal lobe lesions.

### Histopathological examination

**Fig. 6:**
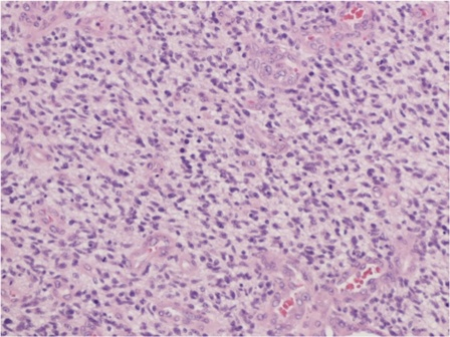
Microphotography of the biopsy specimen revealed highly cellular malignant glial neoplasm, embedded in glial fibrillary background, with mitosis, endothelial proliferation, and necrosis; findings are consistent with glioblastoma (hematoxylin-eosin stain, ×40 magnifications)

**Fig. 7:**
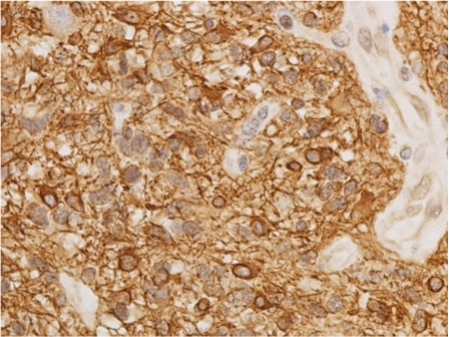
Tumor cells are highlighted with glial acidic fibrillary protein staining (×40)

**Fig. 8:**
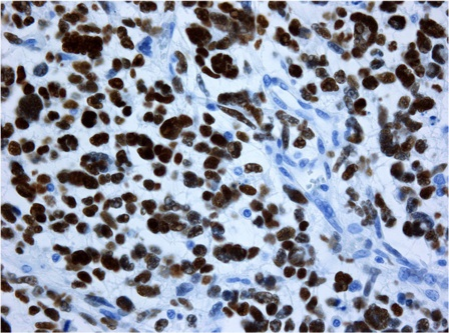
Immunohistochemical staining positive for P53 protein (×40)

**Fig. 9:**
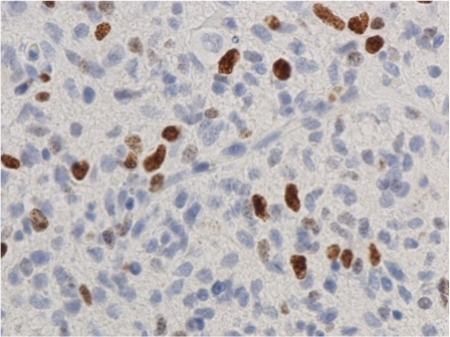
Immunohistochemical staining positive for isocitrate dehydrogenase-1 (×40)

**Fig. 10:**
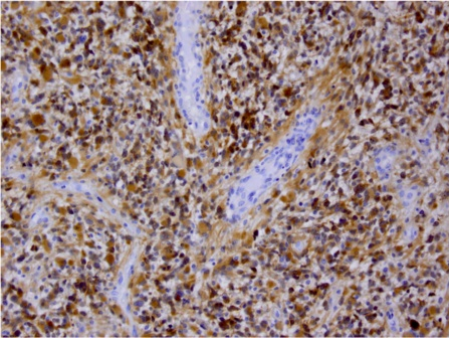
Immunohistochemical staining with Ki-67 with “eye-ball estimation” showing a proliferative index of 5–10 % in focal areas (×40)

H & E stain revealed a highly cellular malignant glial neoplasm with endothelial proliferation and geographical and pseudopalisading necrosis; findings are consistent with glioblastoma (Fig. 6). There were marked mitotic figures which were apparent. The neoplastic cells are embedded in thickened fibrillary stroma, the latter highlighted with glial acidic fibrillary protein (GFAP) (Fig. 7). Tumor cells are positive for P53 (Fig. 8) and isocitrate dehydrogenase (IDH-1) (Fig. 9) immunolabelings. Tumor cells were found to be negative for reticulin and epithelial membrane antigen (EMA). The Ki-67 proliferative index is estimated to be 5–10 % in focal areas (Fig. 10).

The patient had an uneventful postoperative course; he and his family declined any adjuvant chemotherapy and radiotherapy and received palliative care at a rehabilitation center. He died 10 weeks later due to progression of the disease. No postmortem autopsy has been performed.

### Discussion

The development of two or more distinct types of brain tumors is a rare phenomenon associated mostly with radiation exposure or phakomatosis [10, 11]. However, there are scattered reports describing the co-occurrence of two or more histologically different tumors in patients who were not exposed to radiation, nor had phakomatosis [2, 12, 13]. The most commonly reported association is the co-occurrence of meningioma and glioblastoma [2]. The majority of these reports describe a collision where the two tumors co-exist in a single primary lesion or close proximity [2, 13], while cases where the two tumors are located in totally different sites are less frequently reported [14, 15].

Some authors have attributed this co-existence to mere chance [12, 14] giving that meningioma and glioblastoma together account for approximately 51 % of all primary central nervous system tumors. The development of a glioblastoma following the total resection of a meningioma has only been reported twice in the literature [3, 4]. Despite several authors having concluded that this phenomenon was most likely a random statistical coincidence, their different proposed theories have been described in the literatures. Single transduction pathway dysfunction may play an important role in the tumorigenesis of adjacent double tumor. It has been found that the expression of epidermal growth factor receptor (EGFR), platelet-derived growth factor receptor (PDGFR), and vascular endothelial growth factor (VEGF) are involved in this mechanism [16]. Overexpression of EGFR (ErbB1) correlates with enhanced malignant potential of many human tumor types including glioblastoma. The EGFR family of the tyrosine kinase receptor plays an important role in a wide variety of tumors [17]. EGFR family consists of four receptors: (ErbB1/HER), ErbB2 (HER2/neu), ErbB3 (HER3), and ErbB4 (HER4). When the EGFR family members are activated by other ligands, intracellular signaling pathways are triggered which regulate cell division [17]. EGFR family members (EGFR, ErbB2–4) have been evaluated concomitantly in glioma and meningioma. EGFR expression has been reported in 20 % of benign meningioma [17]. The protein expression of the different EGFR family members was predominantly seen in tumor cells in both glioma and meningioma, except for ErbB2. This latter observation could indicate that ErbB2 is involved in tumor angiogenesis of different brain tumors [18].

Furthermore, the P53 pathway dysfunction is generally regarded as keys cause of both glioblastoma and grade I meningioma. Both are found in Li-Fraumeni syndrome. Coincidence as mentioned above has been reported as a reasonable explanation [3, 12, 14]. Another hypothesis is that brain scar created during the operation for the first lesion could have led to the development of glioblastoma [4]. This process is found in areas where tissue repair occurs. The analysis showed that PDGF receptor was overexpressed in both tumors, thereby indicating the oncogenic effects of activated signaling of these receptors. The PDGF-mediated paracrine may induce one tumor from another [16]. Patients with severe head injury were reported to have increased the risk for developing glioma [19]. The fact that the vast majority of head trauma patients do not develop glioma suggests that there must be other predisposing factors involved [6].

A few cases of acute ischemic infarction as the first presentation of glioblastoma have been documented in the literature [7, 8]. In a case series done by Morgenstern et al., the authors have reported that 4.9 % of brain tumor cases were initially misdiagnosed as strokes; over half of these, misdiagnosed cases were glioblastoma [18], while three similar cases to ours where the glioblastoma developed in the territory of previous infarction has only been reported [1, 9, 20]. One report described a patient who developed a glioblastoma 2 years following an MCA ischemic infarction [1]. The second one reported an elderly patient who developed a glioblastoma in the territory of a previous hemorrhagic infarction [9]. López-González et al. reported a case where a patient developed a glioblastoma 7 months following an ischemic stroke [20]. The authors, in this case, have speculated that a subclinical glioblastoma has caused the ischemic injury. This explanation is unlikely in the former two cases, especially when considering that untreated glioblastoma has a median survival rate of 3 months [1, 9].

Our case presents a unique situation where the patient developed a glioblastoma on the background of a residual meningioma and an ischemic infarction. To our knowledge, this is the first report describing such a case, and we postulate, as Zhang et al. have hypothesized, that the development of meningioma and glioma collision tumor is a dynamic process, where one type occurs after the other [13]. Our case together with other reported cases where the two tumors have not appeared at the same time might add further support to this hypothesis of dynamic development [2, 4]. Another factor that could have contributed to the occurrence of the glioblastoma in our patient is the post-infarction tissue repair cascade (post-infarct tissue repair defect). Recent evidence has established parallels between brain tissue repair mechanisms and tumorigenesis [21], in which we postulate that astrogliosis secondary to brain ischemia and genetic mutations may have increased the chance of malignant transformation of glial cells into glioblastoma.

## Conclusions

We have presented a rare and first case where glioblastoma has developed on the background of a residual meningioma and an ischemic infarction. The fact that the vast majority of ischemic stroke patients and those diagnosed with meningioma do not develop glioblastoma makes it obvious that the underlying pathogenic mechanisms are a lot more complex and multi-factorial than the stated malignant transformation theory.

### Consent

Written informed consent was obtained from the patient for publication of this case report and the accompanying images. A copy of the written consent is available for review by the Editor-in-Chief of this journal.

## Abbreviation

EGFR: epidermal growth factor receptor
EMA: epithelial membrane antigen
GFAP: glial acidic fibrillary protein
IDH-1: isocitrate dehydrogenase
PDGFR: platelet-derived growth factor receptor
VEGF: vascular endothelial growth factor
